# Reviewing Interventions to Ensure Management of Cholesterol Levels in Psychiatry Inpatients

**DOI:** 10.1192/bjo.2022.432

**Published:** 2022-06-20

**Authors:** Indrajit Chatterjee, Matthew Cordiner, Naomi Booker

**Affiliations:** 1 Wishaw University Hospital, Wishaw, United Kingdom; 2 The Neurodevelopmental Service for Children and Young People, Newmains Health Centre, Newmains, United Kingdom

## Abstract

**Aims:**

Studies have been done to suggest an increased risk of mortality in patients with mental illness, from cardiovascular diseases. This may be a result of factors ranging from lifestyle choices in the patient group, access to health-care facilities, side-effects of antipsychotic use etc. As a suitable predictor of cardiovascular risk, this audit reviews and attempts to improve the management of cholesterol levels in this patient group based on local trust guidelines.

**Methods:**

116 and 120 patients from general adult psychiatry wards were included in two cycles of the audit respectively. Blood results, discharge letters were obtained from the Clinical portal database; drug prescriptions from the ‘Hospital Electronic Prescribing and Medicines Administration (HEPMA)’ database. As per local trust guidelines, it was verified if ‘ASSIGN’ (indicator of cardiovascular risk developed in Scotland) scores were calculated and a statin was prescribed accordingly, lifestyle modification advice provided or blood results communicated to GP in the discharge letter. An email with a flyer was distributed among doctors with trust guidelines, as intervention after the first cycle of the audit, and the results were presented in internal teaching. This was followed by a reaudit in a few months.

**Results:**

In the first cycle, 85 out of 116 patients had a lipid profile done on admission out of which 29 had abnormal levels without a prescription of statin. 6 patients had their abnormal lipid results mentioned in their discharge letter in the absence of an ASSIGN score calculation or lifestyle modification advice. In the second cycle, it was noted that only 35 patients out of 120 had a lipid profile done on admission and a total of 12 patients had abnormal lipid results without a statin prescription. Only 1 patient had their ASSIGN score calculated and 7 patients had their abnormal lipid results documented to the GP.

**Conclusion:**

Unfortunately, considering both cycles of the audit, only a minority of patients had been managed in accordance with trust guidelines and no significant improvement was noted in the results of the reaudit. The importance of efficient management of cholesterol can be highlighted in a relevant forum and any barriers to change in practice may be explored. QRISK3, an alternative to ASSIGN may be suggested, which includes factors like severe mental illness and atypical antipsychotic use.

